# Chlorambucil *versus* observation after anti-*Helicobacter* therapy in gastric MALT lymphomas: results of the international randomised LY03 trial

**DOI:** 10.1111/j.1365-2141.2008.07486.x

**Published:** 2009-02

**Authors:** Barry W Hancock, Wendi Qian, David Linch, Jean-Charles Delchier, Paul Smith, Ira Jakupovic, Cathy Burton, Robert Souhami, Andrew Wotherspoon, Christiane Copie-Bergman, Carlo Capella, Catherine Traulle, Michael Levy, Sergio Cortelazzo, Andres J M Ferreri, Achille Ambrosetti, Graziella Pinotti, Giovanni Martinelli, Umberto Vitolo, Franco Cavalli, Christian Gisselbrecht, Emanuele Zucca

**Affiliations:** 1 Department of Oncology, Weston Park HospitalSheffield (UKLG); 2 MRC Clinical Trials UnitLondon (UKLG); 3 Haematology, University College of London Cancer InstituteLondon, UK (UKLG); 4 AP-HP, Groupe Henri Mondor-Albert Chenevier, Service d’Hépatologie et de Gastroentérologie, CréteilFrance (GELA); 5 Lymphoma Trials Office, Cancer Research UK and UCL Cancer Trials CentreLondon (UKLG); 6 Department of Histopathology, Royal Marsden HospitalLondon, UK (UKLG); 7 AP-HP, Groupe Henri Mondor-Albert Chenevier, Département de Pathologie, INSERM U841, Université Paris 12Faculté de médecine, IFR10, Créteil, France (GELA); 8 Anatomia e Istologia Patologica, Ospedale di Circolo Fondazione MacchiVarese, Italy (IELSG); 9 Clinical Hematology Department, Centre Hospitalier Lyon SudFrance (GELA); 10 Ematologia e C.T.M.O, Ospedale Centrale di BolzanoBolzano (IELSG); 11 Unità Operativa di Oncologia Medica, Ospedale San RaffaeleMilano (IELSG); 12 Divisione di Ematologia, Policlinico G.B. RossiVerona (IELSG); 13 Unità Operativa di Oncologia Medica, Ospedale di Circolo Fondazione MacchiVarese (IELSG); 14 Istituto Europeo di Oncologia, Divisione di Ematoncologia ClinicaMilano (IELSG); 15 Ospedale Maggiore S. Giovanni Battista, UOA Ematologia OspedalieraTorino, Italy (IELSG); 16 Oncology Institute of Southern Switzerland, BellinzonaSwitzerland (IELSG); 17 Haemato oncology department, INSERM Unité 728, Hospital Saint LouisParis, France (GELA)

**Keywords:** gastric MALT lymphomas, chemotherapy, randomised controlled trial, chlorambucil, observation

## Abstract

Gastric mucosa-associated lymphoid tissue (MALT) lymphomas are uncommon tumours characterised by a tendency to remain localised for long periods. The aetiological association between MALT lymphomas and *Helicobacter pylori* is well established. The role of additional chemotherapy after *H. pylori* eradication in localised MALT lymphomas is unclear. The LY03 trial was designed to establish whether chlorambucil after treatment for *H. pylori* would help prevent recurrence. Patients were treated with antibiotics for *H. pylori* infection. Those with successful eradication of *H. pylori* and no evidence of progression of lymphoma were eligible for randomisation to chlorambucil or observation. Two hundred and thirty-one patients were registered. Ninety-seven percent patients had *H. pylori* eradicated after antibiotics and 59% achieved macroscopically normal gastric mucosa. One hundred and ten patients were randomised. With a median follow-up of 58 months, six patients were dead and 17 had recurrent/progressive disease. The recurrence/progression rates at 5 years were 11% for chlorambucil, and 21% for observation with a difference of 10%, 95% confidence interval (CI) = −9% to 29%, *P* = 0·15. No difference was detected in recurrence/progression-free survival [Hazard Ratio (HR) = 0·96, 95% CI = 0·41–2·2, *P* = 0·91] or overall survival (HR = 1·93, 95% CI = 0·39–9·58, *P* = 0·42). This is the first randomised trial to show there is no good evidence to support that additional single agent chemotherapy to anti*-H. pylori* treatment contributes to prevent recurrence in localised gastric MALT lymphomas.

About 40% of all gastric lymphomas (Koch *et al*, 2005) are indolent and the majority of these are extranodal marginal zone B cell of mucosa-associated lymphoid tissue (MALT) type (Isaacson & Spencer, 1987). MALT lymphomas account for approximately 8% of B-cell lymphomas and are characterised by an indolent natural history and a tendency to remain localised for long periods of time.

The onset of MALT lymphoma in the stomach is usually preceded by *Helicobater pylori* infection and this organism can be found in the gastric mucosa in over 90% of cases (Wotherspoon *et al*, 1991). At the time the study was designed an endoscopic diagnosis of chronic gastritis had often been made before the diagnosis was made histologically. Many patients diagnosed as gastric MALT lymphomas underwent gastrectomy as the primary treatment. For localised low-grade lymphoma confined to the stomach wall, the prognosis was excellent with over 90% of patients surviving 5 years after resection (Ferrucci & Zucca, 2007).

Currently, following the recognition of the aetiological relationship between MALT lymphomas and *H. pylori* (Wotherspoon *et al*, 1991) and the subsequent demonstration of lymphoma regression following successful eradication with antibiotics of the micro organism (Wotherspoon *et al*, 1993; Bayerdorffer *et al*, 1995; Roggero *et al*, 1995), the diagnosis is usually made by endoscopic biopsy. A non-surgical, stomach-conserving approach (i.e. eradication of *H*. *pylori* as the sole initial treatment) is widely employed in the treatment. However, no evidence-based treatment guidelines exist for the management of patients after antibiotic failure and in particular whether there is a role for further adjuvant therapy, such as chemotherapy (Yoon *et al*, 2004; Ferrucci & Zucca, 2007). Some data on the efficacy of chlorambucil as first line treatment in gastric MALT lymphomas has been reported in the older literature. One non-randomised trial by Hammel *et al* (1995) assessed the activity of cyclophosphamide and chlorambucil in low-grade MALT lymphomas, demonstrating 5-year event-free survival and overall survival of 50% and 75% respectively.

The International Extranodal Lymphoma Study Group (IELSG) and United Kingdom Lymphoma Group (UKLG), together with the Groupe d’Etude des Lymphomes de l’Adulte (GELA), conducted a trial to ascertain whether the addition of chlorambucil is of benefit after anti-*H. pylori* therapy in patients with non-progressive gastric MALT lymphomas. No other randomised trials have been performed in gastric MALT lymphomas before.

## Patients and methods

### Patient selection

Patients were eligible for registration if aged 18 years or over with non-resected, partially or completely resected low-grade gastric lymphomas, stage I according to the Blackledge-modified Lugano staging system (Rohatiner *et al*, 1994), and with or without histological evidence of *H. pylori* infection. All cases were subsequently reviewed centrally. Ethical committee approval was obtained for the study and all patients gave informed consent.

### Trial design

Eligible patients were registered and treated with antibiotics according to local practices for *H. pylori* infection. Endoscopies were performed 2–3 months after treatment to assess eradication of *H. pylori*. Confirmation of successful eradication of *H. pylori* was according to local practice as there is usually a good agreement on the value of histological assessment of *H. pylori* eradication. The assessment of tumour response would be performed up to 4–6 months after the organism had been eradicated.

Patients in whom *H. pylori* was successfully eradicated with complete regression of lymphoma were eligible for randomisation. Patients in whom *H. pylori* was successfully eradicated with partial regression or stabilisation of lymphoma might be randomised according to the clinician’s discretion.

Patients were randomised to either observation or chlorambucil. Chlorambucil was chosen because, at the time the study was designed, single-agent chlorambucil was the accepted standard treatment for low-grade Non-Hodgkin lymphomas in most European countries. The drug was given 6 mg/m^2^ daily orally for 14 d, repeated every 28 d for six cycles.

### Pathology review

All gastric biopsies of patients registered were reviewed independently by a panel of pathologists. Histological diagnosis of gastric MALT lymphomas was made according to the criteria defined by Isaacson and Wright (1983) with the presence of an extrafollicular or perifollicular diffuse infiltrate of centrocyte-like cells in the lamina propria with lymphoepithelial lesions. Immunohistochemistry was performed on paraffin sections using antibodies against CD20, CD3 and cytokeratin where appropriate. Presence of *H. pylori* was assessed on modified Giemsa stained sections and, in a proportion of cases, by culture.

### Investigations and response assessment

Investigations prior to randomisation and during follow-up included history, physical examination and routine blood tests. Assessment of tumour response was not made until 4–6 months after eradication of *H. pylori*. Endoscopy was performed every six months for the first 2 years then annually or if there was a recurrence of symptoms. Tumour response was assessed at each post treatment endoscopy with at least eight biopsies. Histological assessment of lymphoma response was made using the scoring system defined by Wotherspoon *et al* (1993). A complete remission (CR) was defined as a patient with endoscopic normalization and a Wotherspoon Score (WS) ≤ 2; a partial remission (PR) was for a patient with endoscopic normalization and a WS > 2; a patient with endoscopic stable disease (SD) and WS > 2 was defined as SD; and patients with extension of the endoscopic lesions and WS > 2 were considered progressive disease (PD). After a CR, disease relapse was defined if a patient had any disease occurred; after a PR or SD, disease progression was defined if the disease got worse.

### Treatment on relapse or progression

Relapse or progression was confirmed histologically where possible and the site of relapse documented. It was recommended that the patient be restaged. If *H. pylori* infection was present, further antibiotics were given. If a CR was then obtained, a further period of observation was undertaken. For patients who failed to achieve CR after further antibiotics, an incomplete response was treated according to local practices (chlorambucil was recommended) in the observation arm. In the chlorambucil arm, an incomplete response was treated with further cytotoxic treatment. The choice was left to the judgement of the clinician. Recommended options included less aggressive chemotherapy, such as further chlorambucil, and more aggressive regimens, such as CHOP (cyclophosphamide, doxorubicin, prednisone, vincristine). The choice of local resection or radiation therapy was also at the discretion of the clinician.

### Statistical considerations

The primary outcome measure was recurrence/progression rate; secondary outcome measures were recurrence/progression-free survival and overall survival. The anticipated endoscopic relapse rate in patients treated for *H. pylori* infection without chlorambucil was 40% at 5 years, meaning a total of 173 patients would be required to demonstrate a reduction to 20% with 5% significance level and 80% power. Planned recruitment was therefore 200 patients randomised.

Recurrence/progression rate was estimated using the Kaplan–Meier approach; patients who died from causes other than lymphoma were censored at the time of death. Recurrence/progression-free survival was defined from the date of randomisation to the date of the first recurrence/progression or date of death from any cause whichever occurred first; at the time of the analysis survivors without disease recurrence/progression were censored at the date of last follow-up. Overall survival was defined from the date of randomisation to the date of death from any cause; at the time of the analysis survivors were censored at the date they were last known to be alive. The log-rank test was applied to compare the Kaplan–Meier curves for recurrence-free survival and overall survival. The relative benefits of chlorambucil on recurrence/progression and overall survival were assessed in an exploratory manner in the subgroup defined by the initial response after antibiotics before randomisation.

All analyses were done on an intention-to-treat basis. All *P* values were two-sided and were considered significant when <0·05. Analyses were performed using sas 9.10.

## Results

### Patients

**Fig 1:**
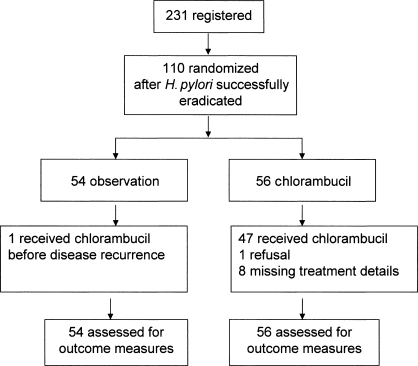
Trial profile.

Between March 1995 and March 2001, a total of 231 patients (132 IELSG, 64 UKLG, 35 GELA) were registered internationally at the UK Lymphoma Trials office (UK LTO) and GELA. One hundred and ten of these patients were subsequently randomised (56 to chlorambucil and 54 to observation) (Fig 1). The trial was stopped early due to slow recruitment influenced, it was thought, by increasing awareness of the long-term successes possible with *H. pylori* eradication.

The baseline characteristics of patients registered are summarised in Table I. There were 231 patients with median age of 62 years of whom 51% were male. The patients had an Eastern Cooperative Oncology Group (ECOG) performance score (PS) of 0–1 in 98% of cases and 97% were stage I. B symptoms were present in 8%. *H. pylori* infection was present in 89% and resection had been undertaken in 7%. Other symptoms at registration included nausea/vomiting (10%), pain/dyspepsia (55%) and anorexia (10%). Associated autoimmune disease was present in 7% (Hashimoto thyroiditis 2%, rheumatoid arthritis 1%, other 4%), and 8% had a second tumour. The most common sites of lymphoma were antrum only (41%), corpus only (24%) and antrum + corpus (16%). There were 3% patients who had the endoscopic appearance of a normal mucosa, while 28% had features of chronic gastritis/hyperaemia, 3% had erosions, 38% had an ulcer, and 31% had other endoscopic appearances or combinations of two or three of the previous findings.

**Table I:** Patients characteristics at registration.

Total	*n* = 231 (%)
Group	
GELA	36 (15)
IELSG	132 (57)
UKLG	64 (28)
Age (years)	
≤60	104 (45)
60–65	31 (13)
>65	96 (42)
Median (range)	62 (20–86)
Male	118 (51)
Site of gastric lymphoma	
Antrum	90 (41)
Corpus	53 (24)
Fundus	20 (9)
Stump	4 (2)
Other	6 (3)
Antrum + corpus	26 (12)
Antrum + fundus	5 (2)
Antrum + other	2 (1)
Antrum + corpus + fundus	9 (4)
Corpus + fundus	5 (2)
Corpus + fundus + other	1 (<1)
Fundus + stump	1 (<1)
Unknown	9
Macroscopic finding: normal mucosa	
No	214 (97)
Yes	7 (3)
Unknown	10
*H. Pylori* infection	
No	26 (11)
Yes	202 (89)
Unknown	3
Previous resection	
No	212 (93)
Partial	9 (4)
Complete	6 (3)
Vagotomy	1 (0)
Unknown	3
Stage	
I	214 (97)
II_1_	6 (3)
Unknown	11
ECOG performance status	
0	163 (76)
1	46 (21)
2	5 (2)
Unknown	17
B symptoms	
No	208 (92)
Yes	17 (8)
Unknown	6
Immune disorder	
No	204 (90)
Yes	22 (10)
Unknown	5
Second tumour	
No	208 (92)
Epithelial	15 (7)
Haematological	2 (1)
Unknown	6

GELA, Groupe d’Etude des Lymphomes de l’Adulte; IELSG, International Extranodal Lymphoma Study Group; UKLG, United Kingdom Lymphoma Group; ECOG, Eastern Cooperative Oncology Group.

Of the 231 registered patients, 97% had *H. pylori* eradicated after antibiotics and 59% had macroscopically normal mucosa. The tumour response rates were CR in 46%, PR in 20%, SD in 30% and recurrence/progression in 8% (Table II). One hundred and ten patients were randomised (56 chlorambucil and 54 observation) with 63 patients in CR, 23 with PR, 15 with SD and nine unknown (Table II). The baseline characteristics of randomised patients were similar to those of all patients at initial registration and were balanced between randomisation arms (Table III). The subsequent analyses focused on randomised patients only.

**Table II:** Response after antibiotics.

			Randomised		
			
	Total (%)	Not randomised	Observation	Chlorambucil	Total
Total	231	121	54	56	110
CR	92 (46)	29	31	32	63
PR	40 (20)	17	11	12	23
SD	59 (29)	44	7	8	15
PD	8 (4)	8	0	0	0
*H. pylori* eradication failure	3 (1)	3	0	0	0
Unknown	29	20	5	4	9

CR, complete remission; PR, partial remission; SD, stable disease/no change; PD, progressive disease.

**Table III:** Patients characteristics at registration for randomised patients.

	Observation	Chlorambucil
		
Total	*n* = 54 (%)	*n* = 56 (%)
Group		
GELA	11	11
IELSG	34	30
UKLG	9	15
Age (years)		
≤60	21 (39)	30 (54)
60–65	10 (19)	9 (16)
>65	23 (43)	17 (30)
Median (range)	63 (28–86)	58 (27–86)
Male	28 (52)	32 (57)
Site of gastric lymphoma		
Antrum	23 (43)	18 (34)
Corpus	10 (19)	18 (34)
Fundus	9 (17)	5 (9)
Stump	0 (0)	1 (2)
Other	0 (0)	1 (2)
Antrum + corpus	6 (11)	6 (11)
Antrum + fundus	1 (2)	2 (4)
Antrum + other	2 (4)	0 (0)
Antrum + corpus + fundus	2 (4)	2 (4)
Corpus + fundus	1 (2)	0 (0)
Unknown	0	3
Macroscopic finding: normal mucosa		
No	51 (96)	54 (100)
Yes	2 (4)	0
Unknown	1	2
*H. Pylori* infection		
No	5 (9)	3 (5)
Yes	49 (91)	53 (95)
Previous resection		
No	50 (93)	50 (91)
Partial	1 (2)	4 (7)
Complete	3 (6)	1 (2)
Unknown	0	1
Stage		
I	52 (98)	53 (100)
II1	1 (2)	0 (0)
Unknown	1	3
ECOG performance status		
0	43 (83)	36 (68)
1	7 (13)	15 (28)
2	2 (4)	2 (4)
Unknown	2	3
B symptoms		
No	54 (100)	49 (91)
Yes	0 (0)	5 (9)
Unknown	0	2
Immune disorder		
No	47 (87)	52 (95)
Yes	7 (13)	3 (5)
Unknown	0	1
Second tumour		
No	50 (93)	48 (89)
Epithelia	4 (7)	4 (7)
Haematological	0 (0)	2 (4)
Unknown	0	2

GELA, Groupe d’Etude des Lymphomes de l’Adulte; IELSG, International Extranodal Lymphoma Study Group; UKLG, United Kingdom Lymphoma Group; ECOG, Eastern Cooperative Oncology Group.

### Treatment received

There was 98% protocol treatment compliance. One patient in the chlorambucil arm did not have the allocated treatment due to the patient’s refusal of chemotherapy. In the observation arm, one patient had chlorambucil 10 months after randomisation without disease recurrence. Chlorambucil was given for a median of 29 weeks (range 3–39).

As chlorambucil was the accepted standard treatment for low-grade Non-Hodgkin lymphomas in most European countries at the time the study was designed, with a well-known safety profile and high tolerability, toxicity data were not collected in detail. In fact, no cases of severe treatment-related toxicity were reported.

### Outcome measures

At the time of analysis, the median (range) follow-up from randomisation was 58 (4–115) months. The events of recurrence/progression or death are summarised in Table IV.

**Table IV:** Summary events for randomised patients.

	Observation	Chlorambucil
		
Total	*n* = 54 (%)	*n* = 56 (%)
Alive without recurrence/progression	43 (80)	45 (80)
Alive with recurrence/progression	9 (17)	7 (13)
Dead without recurrence/progression	1 (2)	4 (7)
Recurrence/progression and dead	1 (2)	0 (0)

**Fig 2:**
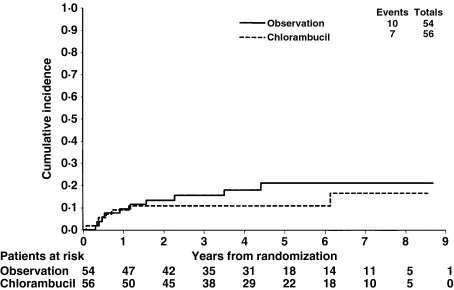
Cumulative incidence rate of recurrence/progression.

A total of 17 patients had disease recurrence/progression (10 in the observation arm and seven in the chlorambucil arm). The 5-year recurrence/progression rates from randomisation were 21% in the observation arm and 11% in the chlorambucil arm, with a difference of 10% and 95% confidence interval (CI) of the difference being −9% to 29%, *P* = 0·15. Figure 2 shows the cumulative incidence rate.

**Fig 3:**
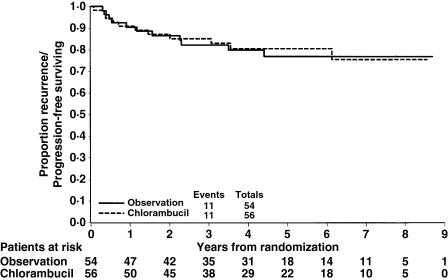
Recurrence/progression-free survival.

Twenty-two patients (11 in each arm) had disease recurrence/progression or died. No difference was detected between the two treatment arms [Hazard Ratio (HR) = 0·96, 95% CI = 0·41–2·2, *P* = 0·91]. The 5-year recurrence/progression-free rate from of randomisation for all randomised patients was 79% (Fig 3).

**Fig 4:**
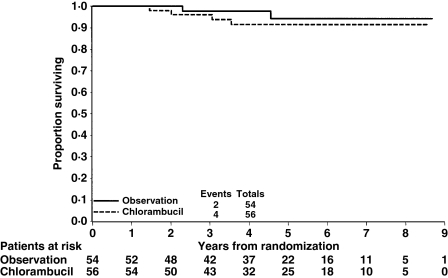
Overall survival.

There were six deaths in total at the time of analysis. Two occurred in the observation arm and four in the chlorambucil arm. The two deaths in the observation arm were due to a myocardial infarction in a patient who had disease progression before death in one patient and to a small cell lung cancer in the other. The four deaths in the chlorambucil arm were due to malignant melanoma (1), cardiac failure (2) and unknown (not lymphoma related) (1). There was no survival difference between the two arms (HR = 1·93, 95% CI = 0·39–9·58, *P* = 0·42). The 5-year overall survival rate from randomisation for all randomised patients was 93% (Fig 4).

There is no evidence that chlorambucil was more or less effective than observation in different responses to antibiotics at randomisation for either recurrence/progression or overall survival.

## Discussion

Localised gastric MALT lymphoma is an indolent disease with characteristic features. Despite abundant literature on histological, clinical and biological features of MALT lymphoma, results of controlled trials to define optimal therapy are limited. Insufficient staging and outdated histological classifications are a major problem of the older reports. Even more recent studies often refer to retrospective series of patients not uniformly staged and treated (Ferrucci & Zucca, 2007). Moreover, the interpretation of residual lymphoid infiltrate in post-treatment gastric biopsies is often difficult and there is a lack of uniform reproducible criteria in the literature for the definition of histological remission (Ferrucci & Zucca, 2007). The Wotherspoon Score (Wotherspoon *et al*, 1993) we used is very useful to express the degree of confidence in the MALT lymphoma diagnosis on small gastric biopsies, it was initially proposed as a simple method to define the histological responses following antibiotic treatment, however, it is sometimes difficult to apply in the evaluation of the response to therapy. Thus, other criteria have been proposed with the aim of providing clinically relevant information to the clinician (Copie-Bergman & Wotherspoon, 2008). These differences in evaluation criteria explain the wide range of the reported remission rates in the literature (Ferrucci & Zucca, 2007).

Our study confirmed that *H. pylori* eradication results in regression of lymphoma in the majority of cases (65% CR/PR, 29% SD and 5% PD). Addition of ‘adjuvant’ chlorambucil conferred no benefit. In the randomised study 5-year recurrence/progression-free survival was similar (77%*versus* 81%) for observed and chlorambucil treated patients. Overall survival was also similar (94%*versus* 92% respectively). In none of the randomised patients on the trial was lymphoma the cause of death and there have been no cases of transformation from low- to high-grade lymphoma. The median follow-up for patients on trial is still only 58 (range 4–115) months; however over one half of the lymphoma relapses (10 in 17 cases) occurred in the first year of follow-up.

Of the original 231 patients registered only 110 (well short of the original target) went on to be randomised. Eleven patients were not randomised due to disease progression and 44 due to inadequate response to antibiotics. Other cases were not randomised despite being eligible – sometimes because of patient refusal, more often clinician reluctance (as initiation of the study preceded the recognition of the excellent prognosis of antibiotic-treated gastric MALT lymphoma).

Indeed, there has been increasing evidence over the years that *H. pylori* eradication as single therapy results in excellent disease control in early-stage gastric MALT lymphoma (Chini *et al*, 2007; Fischbach *et al*, 2007). We are aware that current study was not powered to detect a small difference between two groups. However, it may not be possible in practice to conduct a larger randomised study with time to event outcome measures for this group of patients due to patient population and excellent disease control. In addition, the relapse/progression rate (including in the observational arm) was less than had been anticipated when the trial was designed – this may reflect the fact that the analysis includes only randomised patients who had not progressed after anti-*Helicobacter* therapy, by definition a better prognostic group. Nevertheless, this is the only randomised study in gastric MALT that has been conducted.

In a large German MALT lymphoma study (Stolte *et al*, 2002) 120 patients were followed up endoscopically after anti-*Helicobacter* therapy; 81% achieved CR, 9% PR and 10% were non-responders. In those with complete remission there were nine recurrences, none with local high-grade transformation. With a median follow-up of 4 years, two of the non-responders died of their lymphoma. The others had surgery and/or chemotherapy and in half the lymphoma had become histologically high-grade. With PR, patients had surgery, chemotherapy or, in two cases, surveillance only; there was one death, from a stroke. This series is not, of course, directly comparable to ours where reported follow-up is only for those randomised to either observation or chlorambucil after attaining histological response or stable disease following *H. pylori* eradication.

We have previously reported the clinical and molecular follow-up of these patients following anti-*Helicobacter* therapy (Bertoni *et al*, 2002). Whilst more than one half of patients with MALT lymphoma failed to achieve sustained molecular remission after treatment, this does not appear to be associated with histological relapse, though only long-term follow-up can confirm this (Bertoni *et al*, 2002). Meanwhile, medium term follow-up of those patients proceeding on the randomised study confirmed the excellent prognosis of those who respond to such therapy. We can also conclude that chlorambucil chemotherapy makes no difference to the recurrence/progression of lymphoma. This cannot of course be extrapolated to infer that all chemotherapy will make no difference, however, the gains in terms of increased survival and quality of life would have to be substantial to make the intervention worthwhile. On the other hand, rituximab may be worthy of evaluation in the adjuvant situation (Martinelli *et al*, 2005). Only 10% of patients in this randomised study had gastric resection; none subsequently had further surgery. This confirms current practise where the role for surgery in the treatment for gastric lymphoma is declining (Yoon *et al*, 2004; Ferrucci & Zucca, 2007).

Various markers have been suggested as predictive of response to anti-*Helicobacter* therapy, for example stage of disease, depth of infiltration and t(11:18) translocation (Ruskone-Fourmestraux *et al*, 2001; Wundisch *et al*, 2005). Recently the presence of t(11:18) has been shown to be associated with resistance to oral alkylating agents (Levy *et al*, 2005) but not to rituximab (Martinelli *et al*, 2005). At the time of the start of this trial the association between the translocation and chlorambucil was unknown and this has yet to be analysed in our cohort. Nevertheless, as the translocation is associated with a lack of response to antibiotics (Liu *et al*, 2002) it seems unlikely that its presence may have biased the results of the study because most patients (86/110) had a clear histological regression of the lymphoma at the time of randomisation, with well balanced overall (78% in both arms) and complete (67% in both arms) remission rates.

Based on these early results we can conclude that there is no clear evidence that single agent chemotherapy using chlorambucil contributes to the prevention of recurrence/progression in localised gastric MALT lymphoma after successful treatment with antibiotics. This might be due to the very favourable outcome of the patients in the observation group. This could not be expected at the time the study was designed but nowadays there is increasing evidence that antibiotic treatment allows an excellent long-term disease control and after successful eradication of the micro organism a ‘watch and wait’ policy with regular endoscopies and biopsies appears to be safe even in patients with evidence of residuals of MALT lymphoma (Chini *et al*, 2007; Fischbach *et al*, 2007). On the other hand, this good long-term outcome of many patients with less than complete lymphoma remission should minimize the possible selection bias related to the enrolment in this study of PR and SD patients according to investigator discretion.

Longer follow-up will allow further assessment of time to recurrence/progression and incidence of transformation to high-grade disease. Clinical practice has moved away from surgery over the past 10 years or so (Yoon *et al*, 2004; Bertoni & Zucca, 2005) and this and other studies (Bayerdorffer *et al*, 1995; Roggero *et al*, 1995; Pinotti *et al*, 1997; Koch *et al*, 2005; Wundisch *et al*, 2005) have influenced this change.
